# TLR4 Associated Signaling Disrupters as a New Means to Overcome HERV-W Envelope-Mediated Myelination Deficits

**DOI:** 10.3389/fncel.2021.777542

**Published:** 2021-11-23

**Authors:** Peter Göttle, Kira Schichel, Laura Reiche, Luisa Werner, Annika Zink, Alessandro Prigione, Patrick Küry

**Affiliations:** ^1^Department of Neurology, Medical Faculty, Heinrich-Heine-University, Düsseldorf, Germany; ^2^Department of General Pediatrics, Neonatology and Pediatric Cardiology, Medical Faculty, Heinrich-Heine-University, Düsseldorf, Germany

**Keywords:** myelin repair, multiple sclerosis (MS), oligodendrocyte, toll-like receptor, HERV-W, ENV, mitochondrial dysfunction, endogenous retrovirus

## Abstract

Myelin repair in the adult central nervous system (CNS) is driven by successful differentiation of resident oligodendroglial precursor cells (OPCs) and thus constitutes a neurodegenerative process capable to compensate for functional deficits upon loss of oligodendrocytes and myelin sheaths as it is observed in multiple sclerosis (MS). The human endogenous retrovirus type W (HERV-W) represents an MS-specific pathogenic entity, and its envelope (ENV) protein was previously identified as a negative regulator of OPC maturation—hence, it is of relevance in the context of diminished myelin repair. We here focused on the activity of the ENV protein and investigated how it can be neutralized for improved remyelination. ENV-mediated activation of toll like receptor 4 (TLR4) increases inducible nitric oxide synthase (iNOS) expression, prompts nitrosative stress, and results in myelin-associated deficits, such as decreased levels of oligodendroglial maturation marker expression and morphological alterations. The intervention of TLR4 surface expression represents a potential means to rescue such ENV-dependent deficits. To this end, the rescue capacity of specific substances, either modulating V-ATPase activity or myeloid differentiation 2 (MD2)-mediated TLR4 glycosylation status, such as compound 20 (C20), L48H437, or folimycin, was analyzed, as these processes were demonstrated to be relevant for TLR4 surface expression. We found that pharmacological treatment can rescue the maturation arrest of oligodendroglial cells and their myelination capacity and can prevent iNOS induction in the presence of the ENV protein. In addition, downregulation of TLR4 surface expression was observed. Furthermore, mitochondrial integrity crucial for oligodendroglial cell differentiation was affected in the presence of ENV and ameliorated upon pharmacological treatment. Our study, therefore, provides novel insights into possible means to overcome myelination deficits associated with HERV-W ENV-mediated myelin deficits.

## Introduction

Multiple sclerosis (MS) is an autoimmune disease of the adult central nervous system (CNS) resulting in the loss of mature oligodendrocytes and their myelin sheaths subsequently leading to neurodegeneration (Lassmann, 2018). Efficient remyelination of resulting white matter lesions is rare but can eventually replace lost oligodendrocytes and myelin sheaths as a result of activation and recruitment of resident oligodendroglial precursor cells (OPCs) (Franklin and Ffrench-Constant, 2008; Tripathi et al., 2010). These cells are, therefore, able to restore axonal functions *via* stabilization, protection, electrical insulation, and by providing trophic and metabolic support (Gensert and Goldman, 1997; Simons and Nave, 2015; Franklin and Ffrench-Constant, 2017). Due to the presence of inhibitory regulators within the MS environment, the overall remyelination efficiency remains low and even further declines with disease progression as these precursor cells often fail to generate new oligodendrocytes (Kuhlmann et al., 2008; Kremer et al., 2011). Hence, overcoming differentiation blockades and enhancement of oligodendrogenesis represent a promising strategy for functional recovery (Göttle et al., 2019b; Kremer et al., 2019a; Manousi et al., 2021). Pointing out one specific inhibitor, the envelope (ENV) protein encoded by the human endogenous retrovirus type W (HERV-W) was previously shown by us to interfere with oligodendroglial cell differentiation (Kremer et al., 2013; Göttle et al., 2019a) contributing to the number of MS-related pathological roles of this factor as summarized previously (Küry et al., 2018). Strategies to overcome the negative impact of the ENV protein, activating the toll like receptor 4 (TLR4) signaling pathway on OPCs, have therefore revealed to be potentially interesting in light of the development of specific myelin repair therapies. This was exemplified by the description of KM91104, a vacuolar-ATPase inhibitor, shown to efficiently neutralize ENV-mediated oligodendroglial effects *via* disruption of TLR4 surface expression (Göttle et al., 2019a). Given the widespread expression profile of V-ATPase, a specific (oligodendroglial) prevention of TLR4 activation is difficult to achieve. We therefore addressed to what degree other small molecules are known to modulate TLR4 cell surface expression and activation, e.g., *via* interfering with V-ATPase activity or myeloid differentiation 2 (MD2)-mediated TLR4 glycosylation (Ohnishi et al., 2003; Saitoh and Miyake, 2006), exhibit the capacity to rescue ENV-dependent myelin deficits. The curcumin derivate [1-ethyl-3,5-bis (3,4,5-trimethoxybenzylidene) piperidin-4-one] L48H37, folimycin (concanamycin A) a macrolide antibiotic agent, and the small molecule compound 20 (C20; chalcone derivate that contains the moiety of (E)-4-phenylbut-3-en-2-one) were previously demonstrated to reduce TLR4 cell surface expression in macrophages thus decreasing nitrosative stress reactions and the production of proinflammatory cytokines, respectively (Eswarappa et al., 2008; Wang et al., 2015; Zhang et al., 2016). Applying these substances to OPCs, we indeed found that oligodendroglial cell differentiation and internode formation can be stabilized in the presence of ENV to various degrees. Moreover, as the myelination process is dependent on high metabolic turnover, which is maintained by mitochondria the essential organelles regulating energy production through oxidative phosphorylation (Tepavcevic, 2021), we revealed for the first time unbalanced mitochondrial homeostasis in the presence of the ENV protein likely to contribute to their impaired myelination activity (Barcelos et al., 2019; Maiuolo et al., 2020). Such ENV-dependent changes in mitochondrial integrity/structure could also be counteracted by means of L48H37 or C20. Our study thus sheds light on a plausible correlation between ENV-dependent nitrosative stress inductions affecting oligodendroglial energy homeostasis that can be reversed *via* specific pharmacological stimulation, hence, revealing new subcellular mechanisms that could provide therapeutic targets for upcoming regenerative MS therapies.

## Materials and Methods

### Oligodendroglial Cell Culture

Generation of primary OPCs from postnatal day zero (P0) cerebral rat cortices (Wistar rats of either sex) was performed as previously described (Göttle et al., 2010). The Institutional Review Board (IRB) of the Zentrale Einrichtung für Tierforschung und wissenschaftliche Tierschutzaufgaben (ZETT) at the Heinrich Heine University Düsseldorf has approved all animal procedures under licenses O69/11 and V54/09. Anti-A2B5 staining (Merck Millipore, Darmstadt, Germany; MAB312R RRID:AB_11213098) revealed that the cultures consisted of 98% oligodendroglial cells (data not shown). OPCs were either seeded onto 0.25 mg/ml poly-D-lysine-coated (PDL, Merck Millipore, Darmstadt, Germany) glass coverslips (13 mm) in 24-well plates (for immunocytochemistry; 2.5 × 10^4^ cells/well) or onto 0.25 mg/ml PDL-coated 24-well plates [for quantitative reverse transcription PCR (qRT-PCR); 5 × 10^4^ cells/well] in high-glucose Dulbecco’s Modified Eagle Medium (DMEM)-based Sato medium. After 1.5 h, oligodendroglial cell differentiation was initiated by Sato medium supplemented with 0.5% fetal bovine serum (FBS) (Capricorn Scientific, Palo Alto, CA, United States). The medium was exchanged every 3 days. To determine the physiological reaction from OPCs in exposure to the substances, OPCs were treated in a dose-dependent manner using concentrations 0.1, 0.2, 0.5, 1, 2, and 5 μM for L48H37 (Merck Millipore, Darmstadt, Germany; Cat #: SML1443–L48H37) and C20 (Invivochem, Köln, Germany; Cat #: V4032; CAS #: 111797-22-9) and 0.1, 0.2, 0.5, 1, 2, and 5 nM for folimycin (Merck Millipore, Darmstadt, Germany; Cat#: 344085) respectively. Stock concentrations of L48H37 (4.14 mM), folimycin (10 μM), and C20 (10 mM) were prepared using dimethylsulfoxide (DMSO, Merck Millipore, Darmstadt, Germany), and this solvent was also used as control at equal dilutions. Total 500 μl of prepared solutions were added to the cells containing either substances or DMSO, and final concentrations of L48H38 (0.5 μM), folimycin (0.2 nM), and C20 (0.5 μM) were used. Oligodendroglial cells were treated with 100 ng/ml full-length ENV protein in a differentiation medium as previously described (Kremer et al., 2013; Göttle et al., 2019a). Recombinant HERV-W ENV protein was produced by Protein’eXpert (Grenoble, France) according to quality control specifications of GeNeuro SA (Lyon, France) and kindly provided by Dr. Hervé Perron (GeNeuro SA). Endotoxin levels were below the detection limit (<5 EU = ml) as measured by the Limulus amebocyte lysate test. Control experiments were conducted using recombinant protein buffer preparation [20 mM histidine, 5% (w/v) sucrose, and 0.01% (w/v) polysorbate 20, pH 6.0]. ENV protein and buffer were applied at equal dilutions in the differentiation medium (DMEM-based Sato-medium with 0.5% FCS). The control treatment and the treatment with the substance were only diluted in differentiation medium. The ENV treatment and the combination of ENV with the substances were diluted in the differentiation medium containing ENV. The following combinations were used: (a) recombinant protein buffer; (b) recombinant ENV protein; (c) recombinant ENV and 0.5 μM L48H37; (d) recombinant ENV and 0.2 nM folimycin; and (e) recombinant ENV protein and 0.5 μM C20.

### RNA Preparation, cDNA Synthesis, and qRT-PCR

Total RNA purification from cultured cells was lysed using 350 μl RLT lysis buffer (Qiagen, Hilden, Germany) supplemented with β-mercaptoethanol (1:100, Merck Millipore, Darmstadt, Germany), and total RNA was purified using the RNeasy Mini Kit (Qiagen, Hilden, Germany) according to manufacturer instructions, such as DNase digestion. Before qRT-PCR, reverse transcription with 250 ng RNA [measured using a NanoDrop ND 1000 (Peqlab, Erlangen, Germany)] was done using the High-Capacity cDNA Reverse Transcription Kit (ThermoFisher Scientific, Darmstadt, Germany). Gene expression levels were determined on a 7900HT sequence detection system (ThermoFisher Scientific, Darmstadt, Germany) applying SybrGreen universal master mix (ThermoFisher Scientific, Darmstadt, Germany). For sequence detection, the following forward (fwd) and reverse (rev) primers, generated *via* PrimerExpress 2.0 software (Applied Biosystems, Waltham, MA, United States), were used with ornithine decarboxylase (ODC) and glyceraldehyde 3-phosphate dehydrogenase (GAPDH) serving as reference genes: r-iNOS_fwd: CTCAGCACAGAGGGCTCAAAG, r-iNOS_rev: TGCACCCAAACACCAAGGT, r-IL-6_fwd: GTT GTGCAATGGCAATTCTGA, r-IL-6_rev: TCTGACAGTGCAT CATCGCTG, r-IL-1ß_fwd: GAAACAGCAATGGTCGGGAC, r-IL-1ß_rev: AAGACACGGGTTCCATGGTG, r-TNF-α_fwd: AGCCCTGGTATGAGCCCATGTA, r-TNF-α_rev:CCGGACTC CGTGATGTCTAAGT, r-TLR4_fwd: CTGGGTTTCTGCT GTGGACA, r-TLR4_rev: AGGTTAGAAGCCTCGTGCTCC, r-Ldha_fwd: CTGTGTGGAGTGGTGTGAATGTC, r-Ldha_rev: CAGCTGCGGGTTCAGAACT, r-myc_fwd: CTTCCCCTA CCCGCTCAAC, r-myc_rev: GTGGAATCGGACGAGGTACAG, ppargc1a_fwd: TGAAGAGCGCCGTGTGATT, r-ppargc1a_rev: TTCTGTCCGCGTTGTGTCA, r-ppargc1b_fwd: GGAAAAGG CCATCGGTGAA, ppargc1b_rev: GCTCATGTCACCGGA GAGATTT, r-pparg-1_fwd: CCCACCAACTTCGGAATCAG, r-ppar-γ-1_rev: GGAATGGGAGTGGTCATCCA, r-pdpk1-1_ fwd: AATGGTGAGGTCCCAGACTGA, CCTGCTAACACCAC TAGGAATGC, GAPDH_fwd: GAACGGGAAGCTCACTGGC, GAPDH_rev: GCATGTCAGATCCACAACGG ODC_fwd: GGT TCCAGAGGCCAAACATC, ODC_rev: GTTGCCACATTGAC CGTGAC. Relative gene expression levels were determined according to the ΔΔCt method (ThermoFisher Scientific, Darmstadt, Germany). All measurements were done in duplicates; generated from *n* = 4 independent experiments, and data are shown as mean values ± SEM.

### Myelinating Co-cultures

Dissociated neuron/oligodendrocyte co-cultures were obtained from embryonic day 16 (E16) rat cerebral cortex (Wistar rats of either sex) according to Pang et al. (2012) and as previously published by us (Göttle et al., 2015). Cortical cells were plated on 15-mm poly-D-lysine (0.1 mg/ml) coated cover slips (65,000 cells per cover slip) and kept in myelination medium consisting of N2 and neurobasal medium (ThermoFisher Scientific, Darmstadt, Germany; ratio 1:1), such as NGF (50 ng/ml) and NT-3 (10 ng/ml) (both R&D Systems, Minneapolis, MN, United States). The day of primary culture was defined as day one *in vitro* (DIV1). After 10 days *in vitro* (DIV10) insulin was excluded, and the ratio of the insulin-free N2 to neurobasal medium, such as B27 supplement (ThermoFisher Scientific, Darmstadt, Germany), was adjusted to 4:1. This myelination medium was further supplemented with 60 ng/ml tri-iodo-thyronine (T3, Merck Millipore, Darmstadt, Germany). Final concentrations of individual N2 medium components (DMEM-F12 based, high glucose; ThermoFisher Scientific, Darmstadt, Germany) were insulin (10 μg/ml), transferrin (50 μg/ml), sodium selenite (5.2 ng/ml), hydrocortisone (18 ng/ml), putrescine (16 μg/ml), progesterone (6.3 ng/ml), biotin (10 ng/ml), *N*-acetyl-L-cysteine (5 μg/ml) (all Merck Millipore, Darmstadt, Germany), bovine serum albumin (0.1%, Roth, Karlsruhe, Germany), and penicillin–streptomycin (50 units/ml, ThermoFisher Scientific, Darmstadt, Germany). At DIV30, cover slips were washed with PBS, fixed with 4% paraformaldehyde, and processed for immunofluorescent staining. At the onset of myelination DIV17, cultures were treated for other 9 days with myelination medium supplemented with ENV in the presence or absence of L48H37, folimycin, or C20 until DIV26 according to Göttle et al. (2015). Myelinating oligodendrocytes were identified as previously described (Göttle et al., 2015, 2018, 2019a).

### Immunostaining

Fixed cells were permeabilized with PBS containing 0.01% Triton X-100 (Merck Millipore, Darmstadt, Germany), and unspecific staining was blocked with 10% normal goat serum or donkey serum (Merck Millipore, Darmstadt, Germany) for 40 min as established previously (Göttle et al., 2010). Cells were then incubated with primary antibodies in PBS with 10% normal goat serum or donkey serum overnight at 4°C using the following dilutions: rat anti-myelin basic protein (MBP; 1/250, Bio-Rad, Munich, Germany; Cat# MCA409S RRID:AB_325004), mouse anti-adenomatosis polyposis coli (CC1; 1:250, GeneTex Cat# GTX16794, RRID:AB_422404), rabbit anti-cleaved caspase-3 (CC-3; 1/500, Cell Signaling Technology Cat# 9661, RRID:AB_2341188), and rabbit anti-TLR4 (1/1,000; Abcam Cambridge, United Kingdom; Cat# ab13556, RRID:AB_300457). Mitochondrial staining was performed by loading alive OPCs, after 24 h treatment, with 50 nM Mitotracker Red CMX-Ros (ThermoFisher Scientific, Darmstadt, Germany) in serum-free Sato medium for 20 min at 37°C, followed by fixation and phalloidin labeling. Thus, cover slips were incubated with 50 μg/ml phalloidin-fluoresceinisothiocyanate (Merck Millipore, Darmstadt, Germany) for visualization of cells. Fixed co-cultures were blocked with PBS containing 0.5% Triton X-100 and 2% normal goat serum and then incubated overnight in 0.1% Triton and 2% normal goat serum containing rat anti-MBP (1/250, Bio-Rad, Munich, Germany; Cat# MCA409S RRID:AB_325004), and rabbit anti-neurofilament (NF, 1:1,000, Abcam, Cambridge, United Kingdom Cat# ab8135, RRID:AB_306298). After 24 h, cover slips were washed with PBS and then incubated with secondary antibodies in PBS (diluted 1/500) for 2 h conjugated to goat anti-rat Alexa Fluor 488 (ThermoFisher Scientific, Darmstadt, Germany; Cat# A-11006 RRID:AB_2534074), goat anti-rabbit Alexa Fluor 405 (ThermoFisher Scientific, Darmstadt, Germany; Cat# A31556 RRID:AB_221605), goat anti-mouse Alexa Fluor 594 (ThermoFisher Scientific, Darmstadt, Germany; Cat# A11005 RRID:AB_10561507), goat anti-rabbit (ThermoFisher Scientific, Darmstadt, Germany; Cat# A11037 RRID:AB_10561549), or goat anti-rat (ThermoFisher Scientific, Darmstadt, Germany; Cat# A-11007, RRID:AB_10561522). Nuclei were stained with 4′,6-diamidin-2-phenylindol (DAPI, Roche, Basel, Switzerland). Images (20×; Zeiss Axionplan2 microscope) were captured using the same light intensity, and filters for all images were compared and processed with Axiovision 4.2 software (Zeiss, Jena, Germany; RRID:SciRes_000111). The analysis was done using Java software (ImageJ, RRID:SCR_003070,467/Wright Cell Imaging Facility, RRID:SCR_003070,471). Immunopositive cells were counted in nine randomly chosen fields per coverslip. Two coverslips were used per condition. The total number of cells per field was determined *via* DAPI staining. For quantification, the number of immune-positive cells was compared to the total cell number and expressed as a percentage [mean ± SEM]. To compare TLR4 surface, expression cells were subjected to confocal laser scanning microscopy using a confocal LSM microscope 510 (CLSM 510, Zeiss, Jena, Germany). The fluorescence intensity across the plasma membrane was detected by using Zen 2012 software (Zeiss) according to Ohgaki et al. (2017).

### Proximity Ligation Assay

Direct protein/protein interactions were investigated by means of a proximity ligation assay (PLA) [(Soderberg et al., 2006) using Duolink *in situ* Red Starter Kit Mouse/Rabbit (Merck Millipore, Darmstadt, Germany] as previously described (Göttle et al., 2015). Cells were rapidly washed with ice-cold PBS and then fixed at room temperature for 10 min in 4% PFA. Cells were further rinsed 3 min × 3 min with PBS containing 0.1% Triton X-100 (Merck Millipore, Darmstadt, Germany) and then washed 3 min × 2 min with 0.05% Tween 20 (Merck Millipore, Darmstadt, Germany) in TBS to allow permeabilization. Then one droplet (40 μl) of Duolink II blocking solution was added to each cover slip and incubated in a preheated humidity chamber for 1 h at 37°C. Next, the blocking solution was tapped off, and cells were incubated for 1 h at 37°C with primary antibodies anti-TLR4 (1/1000; ab13556, Abcam Cambridge, United Kingdom; Cat# ab13556, RRID:AB_300457), mouse anti-ENV (1/500; GNmAB03 provided by GeNeuro; and as established in Kremer et al. (2013), Göttle et al. (2019a), and Kremer et al. (2019b). Duolink anti-rabbit PLUS and anti-mouse MINUS secondary antibodies and Red Detection Reagents were used, and antibody incubation, ligation, amplification, and washing steps were performed according to the manual of supplier. Cover slips were then incubated with 50 μg/ml phalloidin-fluoresceinisothiocyanate (Merck Millipore, Darmstadt, Germany) for visualization of cells, dried, mounted using Duolink Mounting Media with DAPI, and analyzed using Axiovision 4.2 software (Zeiss) and image processing and analysis in Java software (ImageJ, Wright Cell Imaging Facility).

### Statistical Analysis

Data are presented as mean ± SEM. Graphs and statistical analysis were performed using Excel and the GraphPad Prism 8.0.2 software (GraphPad Prism, San Diego, CA, United States; RRID: SCR_002798). Shapiro–Wilk normality test was used to assess the absence of the Gaussian distribution of all datasets. To determine statistical significance for normally distributed data sets, one-way ANOVA with Turkey post-test for multiple comparisons was applied to compare three or more groups. For data sets not passing the Shapiro–Wilk normality test, Kruskal–Wallis test with Dunn’s post-test was applied. Statistical significance thresholds were set as follows: *p < 0.05, ^**^p < 0.01, ^***^p < 0.001 and *n* represents the number of independent experiments.

## Results

Since it was recently demonstrated that L48H37, folimycin (concanamycin A), and C20 are able to interfere with TLR4 signaling (Eswarappa et al., 2008; Wang et al., 2015; Zhang et al., 2016), hence, decreasing stress reaction within macrophages, we hypothesized that these substances could also stabilize and/or rescue oligodendroglial homeostasis and differentiation in the presence of ENV protein. To this end, we investigated the effect of the given substances on purified postnatal primary rat OPCs regarding differentiation dynamics, myelin sheath generation, and mitochondrial integrity.

### Tolerated Dosage of L48H37, Folimycin, and C20 for Rat Oligodendroglial Precursor Cells

**FIGURE 1:**
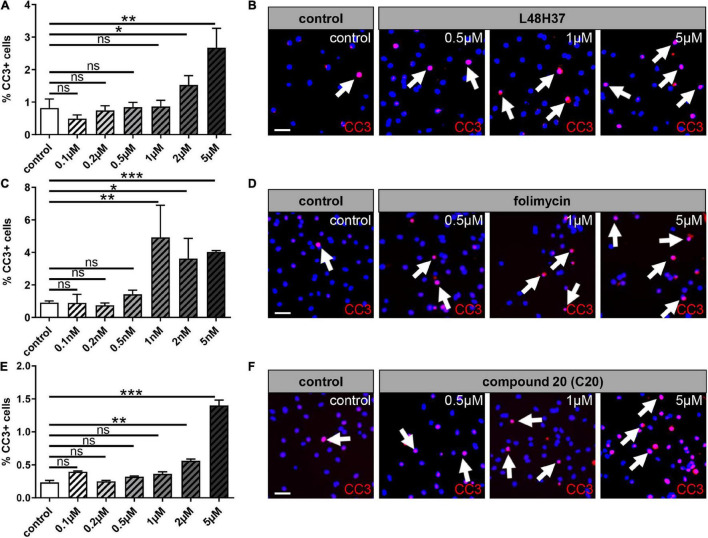
Oligodendroglial cell death rates upon L48H37, compound 20, and folimycin treatment. Immunocytochemical staining by means of cleaved caspase 3 (CC-3) within OPCs after 1 day revealed high doses of L48H37, folimycin, and compound 20 led to an increase in the percentage of apoptotic OPC as judged by the expression of activated CC-3 **(A,C,E)**. DMSO was used as a control and was applied at equal dilutions. Representative pictures show CC3 positive cells marked *via* immunocytochemical staining and DAPI-stained nuclei **(B,D,F)**. Arrows represent CC3 positive cells. Scale bar: 20 μm. Data are shown as mean values (± SEM) and derived from *n* = 3 experiments. Statistical significance was calculated using one-way ANOVA with Tukey post-test (ns, not significant, *p < 0.05, **p < 0.01, and ***p < 0.001). DMSO, dimethylsulfoxide; DAPI, 4′,6-diamidin-2-phenylindol.

To determine functionally relevant cell-specific concentrations, cell death of primary cultured OPCs was investigated in a dose-dependent manner by the detection of activated CC-3 expression. Dilution stock concentrations of L48H37 (4.14 mM), folimycin (10 μM), and C20 (10 mM) were performed, and primary OPCs were stimulated with different concentration series regarding L48H37 and C20 (0.1, 0.2, 0.5, 1, 2, and 5 μM) and folimycin (0.1, 0.2, 0.5, 1, 2, and 5 nM) for 1 day (Figure 1). Immunofluorescent staining for CC-3 revealed that high concentrations (above 0.5 μM for L48H37 and C20; Figures 1A,B,E,F) and (above 0.5 nM for folimycin; Figures 1C,D) substantially increased the degree of apoptotic OPCs. Therefore, for subsequent stimulation experiments, concentrations with low apoptosis rates were used: 0.5 μM for L48H37 (apoptotic rate ≤ 0.85%), 0.2 nM for folimycin (apoptotic rate ≤ 0.75%), and 0.5 μM for C20 (apoptotic rate ≤ 0.3%).

### Envelope Induces the Expression of Proinflammatory Factors and Can Be Reversed by L48H37, Folimycin, and C20

**FIGURE 2:**
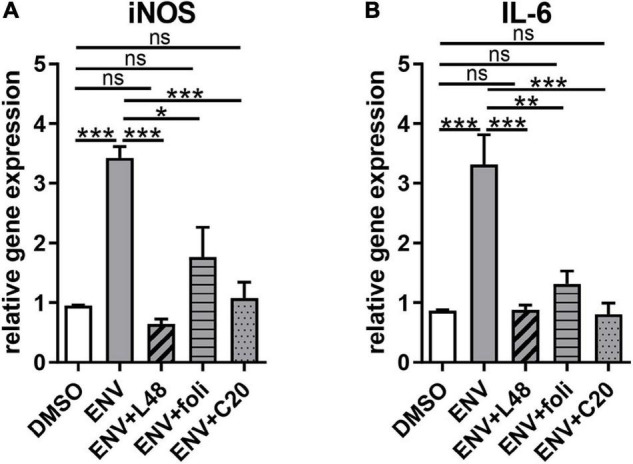
Gene expression alterations due to proinflammatory effects of the recombinant envelope protein (ENV) on cultured rat oligodendroglial precursor cells. **(A,B)** Quantitative RT-PCR after 1d revealed that upon ENV protein stimulation a significant upregulation of *iNOS* and *IL-6* transcript levels occurred, which was compensated to control transcript levels upon L48H37 or compound 20 treatment. Data are shown as mean, and error bars represent SEM. A number of experiments: *n* = 4. Statistical significance was calculated using one-way ANOVA with Tukey post-test (ns, not significant, *p < 0.05, **p < 0.01, and ***p < 0.001). iNOS, inducible nitric oxide synthase; IL-6, interleukin-6.

Given that ENV-mediated activation of TLR4 leads to the induction of proinflammatory cytokine expression, nitrosative stress, and a subsequent reduction in myelin protein expression (Kremer et al., 2013, 2015; Göttle et al., 2019a), it was of interest to investigate the capacity of L48H37, folimycin, and C20 to reverse ENV-dependent gene inductions. To this end, gene expression analysis of OPCs 1 day after treatment was conducted by means of qRT-PCR. ENV stimulation led to a proinflammatory reaction, as transcript levels of *inducible nitric oxide synthase* (*iNOS)* and *interleukin-6 (IL-6)* were upregulated (Figures 2A,B). Of note, L48H37, folimycin, and C20 were able to repress their expression in the presence of ENV at baseline levels.

### Rescue of ENV-Dependent Oligodendroglial Differentiation Deficits by L48H37, Folimycin, and C20

**FIGURE 3:**
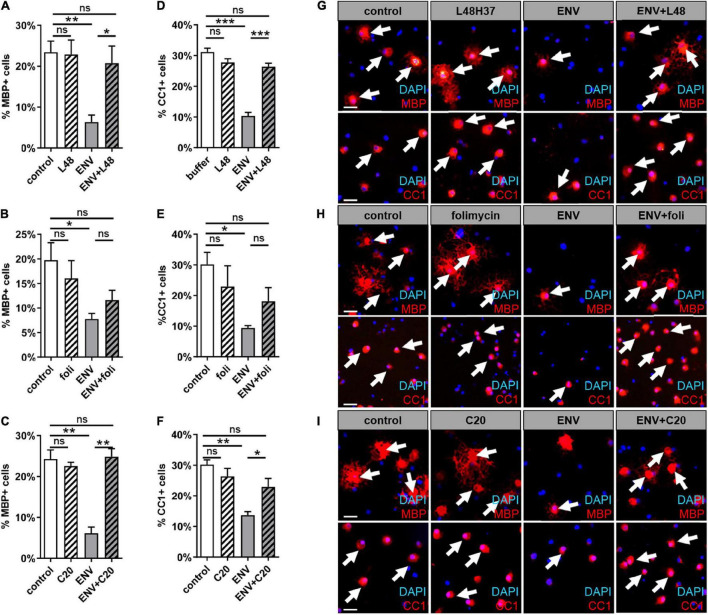
MBP and CC1 expression during spontaneous oligodendroglial precursor cell differentiation upon stimulation with ENV in combination with L48H37, compound 20, or folimycin. **(A–F)** Determination of the percentage of MBP- positive cells upon exposure to protein buffer, to respective substance, to ENV (100 ng/ml) protein or to ENV protein (100 ng/ml) plus respective substance for 3 days. The concentrations for L48H37 (0.5 μM), folimycin (0.2 nM), and C20 (0.5 μM) were determined in the previous CC3 experiment. The protein buffer and the condition with the substance only were diluted in DMSO and were applied at equal dilution. **(G–I)** Representative pictures demonstrate MBP positive OPCs marked *via* immunocytochemical staining (red; indicated through arrows) and DAPI stained nuclei (blue). Scale bar: 50 μm. A number of experiments: *n* = 4. Statistical significance was calculated using one-way ANOVA with Tukey post-test **(A,C,D–F)** and **(B)** Kruskal–Wallis test with Dunn’s post-test (ns, not significant, *p < 0.05, **p < 0.01, and ***p < 0.001). DAPI, 4′,6-diamidin-2-phenylindol; MBP, myelin basic protein; OPCs, oligodendroglial precursor cells; DMSO, dimethylsulfoxide; ENV, envelope protein.

Considering our previous findings demonstrate that the ENV-mediated differentiation/myelination blockade can be overcome by molecules, such as KM91104, which disturbs TLR4 signaling. We were wondering whether the substances L48H37, folimycin, and C20, all of which are known to affect macrophage TLR4 signaling (Eswarappa et al., 2008; Wang et al., 2015; Zhang et al., 2016), could also maintain the differentiation capacity of OPC. For functional examination of the selected substances, we treated primary OPCs with L48H37, folimycin, or C20 and determined differentiation-related expression of myelin and late maturation markers, such as MBP and adenomatosis polyposis coli (CC1) protein expression after 3 days by means of immunofluorescent staining (Figure 3). Substance treatment alone did not significantly affect MBP- or CC1 positivities, but a clear rescue of the diminished expression in presence of the ENV protein could be observed in cells treated with L48H37 or C20 for both markers MBP (Figures 3A,C,G,I) and CC1 (Figures 3D,F,G,I) returned to baseline levels. In contrast, no significant rescue reaction was detected in folimycin treated cells.

### Rescue of ENV-Dependent Myelination Deficits by L48H37 and C20 but Not by Folimycin

**FIGURE 4:**
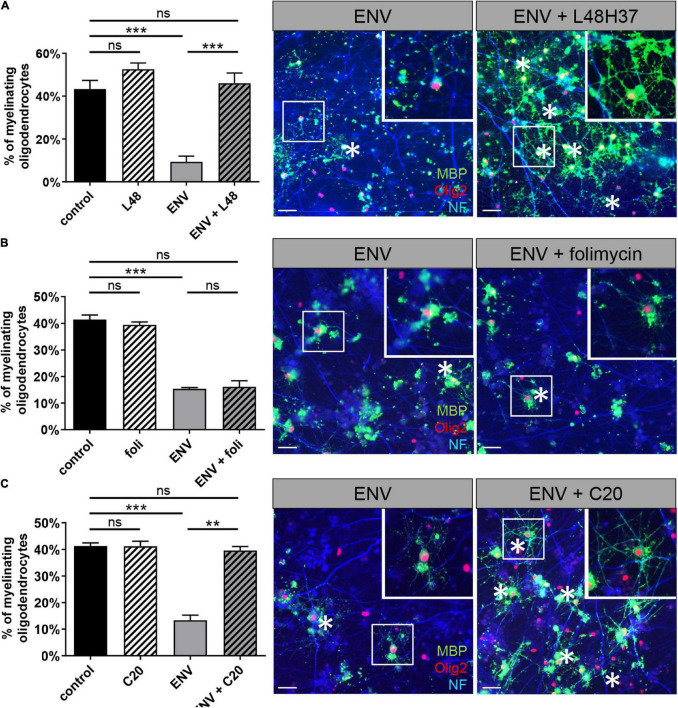
L48H37 and compound 20-mediated rescue of ENV-dependent oligodendroglial myelinating deficits. Quantification of MBP-positive oligodendrocytes (at DIV26) upon ENV protein or ENV protein plus L48H37 **(A)**, folimycin **(B)** or compound 20 **(C)** exposure, starting at DIV17. Representative *in vitro* myelinating co-cultures displaying the rescue of myelinating oligodendrocytes (asterisks; MBP immunostaining signals in green) by means of substance application in presence of ENV protein (axons are visualized by means of NF staining in blue, cell nuclei are marked by Olig2 in red). Scale bars, 50 μM. Inserts in the top right corners correspond to enlarged fields marked by the white squares. Data are shown as mean as values, error bars represent SEM. Number of experiments: *n* = 3. Statistical significance was calculated using one-way ANOVA with Tukey post-test (ns, not significant, **p < 0.01, ***p < 0.001). DAPI, 4′,6-diamidin-2-phenylindol; MBP, myelin basic protein; OPCs, oligodendroglial precursor cells; DMSO, dimethylsulfoxide; ENV, envelope protein.

Next, it was important to extend our findings onto the myelination process (Figure 4). To this end, mixed rat neuron/glia co-cultures were grown for 17 days and then treated for another 9 days with a myelination medium with or without the addition of recombinant ENV protein. These co-cultures were then co-treated with L48H37, folimycin, C20, or control buffer during the entire myelination period as previously described for KM91104 (Göttle et al., 2019a). After a total duration of 26 days in culture, the percentage of MBP positive oligodendrocytes exhibiting a myelinating phenotype (i.e., featuring internodes) was determined based on the total number of Olig2-positive cells. This analysis revealed a full rescue of the degree of myelinating oligodendrocytes in the presence of ENV protein by L48H37 (Figure 4A) and C20 (Figure 4C), but not folimycin, indicating that the ENV-mediated deficiency of this critical neuron/glia interaction can be specifically modulated.

### L48H37 and C20 Affect TLR4 Surface Presentation on Oligodendroglial Precursor Cells

**FIGURE 5:**
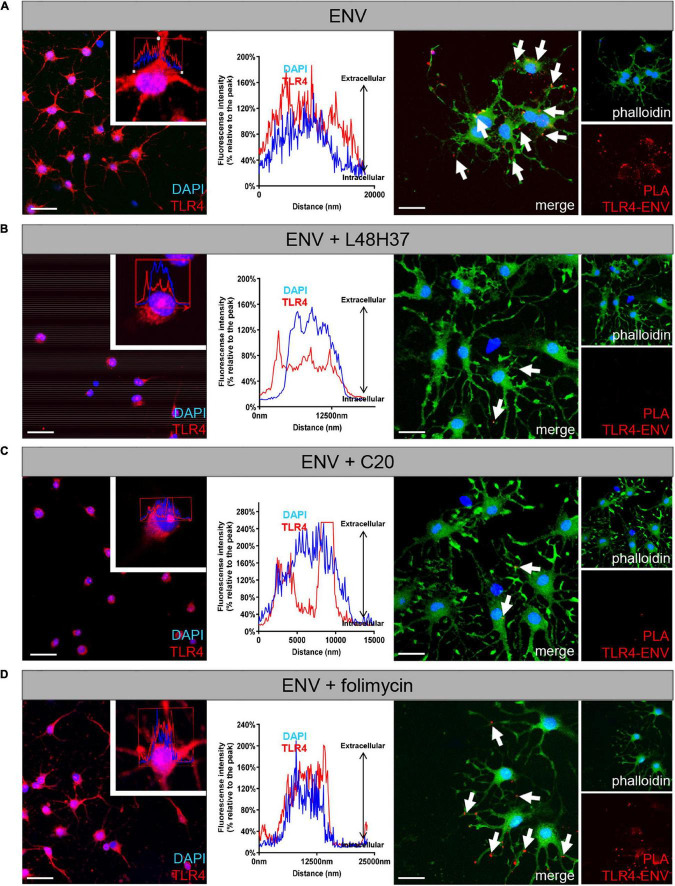
Exposure of L48H37 and compound 20 to oligodendroglial cells affects TLR 4 surface expression. Confocal laser scanning microscopy-based intensity fluorescent peak detection for DAPI (blue) and TLR4 (red) across the cell. Experimental determination of TLR4 surface expression (red) as compared to nuclear DAPI signals following ENV protein exposure only **(A)**, in the presence of L48H37 **(B)**, in the presence of folimycin **(C)** or in the presence of compound 20 **(D)** after 1 day. L48H37 and compound 20 treated cells showed an interior distribution pattern of the receptor molecules, as compared to cells treated with ENV only or in the presence of folimycin where TLR4 was distributed over the entire surface. The normalized fluorescence intensities (% relative to the peak) of TLR4 were plotted against the distance of the analyzed area. Proximity ligation assay (PLA) of oligodendroglial cells after 1 day in culture, positive PLA events (red dots) are indicated by arrows. Positive events could be detected upon binding of ENV protein to the TLR4 receptor following exposure to recombinant ENV protein. No PLA events were detectable in the presence of ENV accompanied by L48H37 or compound 20 treatment. Phalloidin-fluorescein isothiocyanate was used to visualize cell bodies and processes. Scale bars, 30 μM. DAPI, 4′,6-diamidin-2-phenylindol; ENV, envelope protein; PLA, proximity ligation assay.

Given the observed and specific rescue effects of L48H37 and C20 in terms of the ENV-mediated oligodendroglial differentiation blockade, it was of considerable interest to see whether TLR4 surface presentation can be modulated by these three substances (Figure 5). TLR4 surface expression was analyzed after 24 h of substance treatment by means of laser scanning microscopy (LSM). Herein, peak/intensity detection across the cell revealed that the fluorescence peak of TLR4 signal is shifted toward the interior compared to DAPI indicating a profound decrease in TLR4 surface expression in presence of ENV in combination with L48H37 (Figure 5B) or C20 (Figure 5C) as opposed to ENV treatment only (Figure 5A), or when folimycin was applied (Figure 5D). To further confirm that TLR4 surface localization was abolished, we examined physical interactions between TLR4 and ENV proteins *in situ*, using the PLA (Soderberg et al., 2006; Göttle et al., 2015). To this end, OPCs were stimulated with ENV protein in the absence or presence of either L48H37, folimycin, or C20, fixed and subjected to PLA staining procedure. Upon ENV treatment of control cells, positive PLA events reflecting direct TLR4/ENV binding events on the cells surface (distinct red fluorescent spots) could be observed (Figure 5A, right side of the composite Figure). Of note, no direct TLR4/ENV interactions (hence red dots) could be detected in L48H37 and C20 treated cells (Figures 5B,C) whereas folimycin-treated cells apparently still allowed a physical ENV/TLR4 interaction (Figure 5D).

### Envelope Mediated Mitochondrial Changes in Oligodendroglial Cells

**FIGURE 6:**
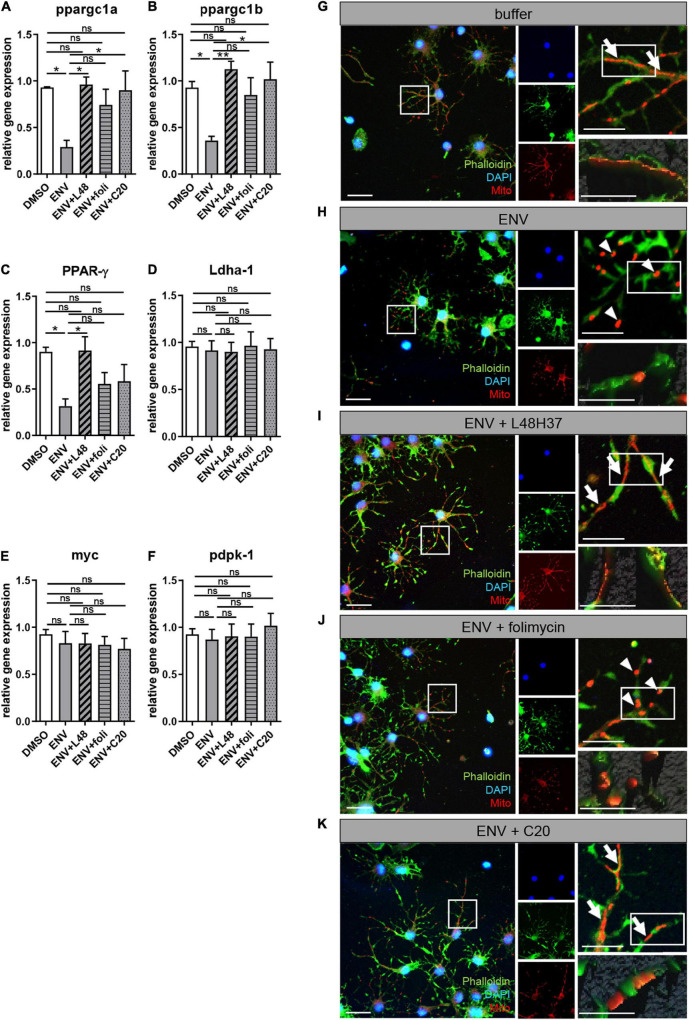
ENV-mediated mitochondrial changes in oligodendroglial cells are rescued upon L48H37 or compound 20 treatment. **(A–F)** Quantitative RT-PCR after 1d of *PPAR-γ, ppargc1a*, *ppargc1b, Ldha-1*, *myc*, or *pdpk-1* revealed that ENV protein exposure led to a significant downregulation of genes related to mitochondrial homeostasis that was reversed upon L48H37 or compound 20 treatment **(A–C)**. Data are shown as mean, and error bars represent SEM. The number of experiments: *n* = 4. Statistical significance was calculated using one-way ANOVA with Tukey post-test (ns, not significant, *p < 0.05, **p < 0.01, and ***p < 0.001). **(G–K)** fluorescence micrographs of Mitotracker labeled mitochondria (red) in phalloidin (green) stained oligodendroglial cells exposed for 1 day to recombinant buffer **(G)**, ENV protein **(H)**, ENV protein and L48H37 **(I)**, ENV protein and folimycin **(J)** or ENV protein and compound 20 **(K)**. Scale bars for overview and upper images 50 μM. Scale bars for the detailed remaining images 20 μM. Elongated mitochondrial shape **(G,I,K)** is marked by arrows whereas fragmented mitochondrial **(H,J)** is indicated by arrowheads. ENV, envelope protein.

Since a balanced mitochondrial function is key for successful oligodendroglial cell differentiation to occur (Schoenfeld et al., 2010) and recent studies associated mitochondrial dysfunction with neurological diseases, such as MS (Lan et al., 2018; Barcelos et al., 2019), we investigated whether ENV exposure affects mitochondria. We treated primary OPCs with L48H37, folimycin, or C20 and determined the expression of genes related to mitochondrial homeostasis after 1 day in the absence and presence of ENV protein (Figure 5). A significant downregulation of *peroxisome proliferator-activated receptor gamma coactivator 1 alpha* (*ppargc1a*), *peroxisome proliferator-activated receptor gamma coactivator 1 beta* (*ppargc1b*), and *peroxisome proliferator-activated receptor gamma* (*PPAR-γ*) was determined upon exposure to ENV protein, all of which encode for major regulators of mitochondrial biogenesis (Corona and Duchen, 2016; Yeligar et al., 2018). These transcript levels could essentially be rescued when OPCs were treated with the L48H37 and C20 generating statistically significant rescue values and with folimycin in failing to do so (Figures 6A–C). The expression of genes involved in cellular energy metabolism, such as *lactate dehydrogenase A* (*Ldha-1*), *proto-oncogene MYC Box 6* (*myc*), or *3-phosphoinositide-dependent protein kinase* (*pdpk-1*), was affected neither by the ENV protein nor the three substances (Figures 6D–F), suggesting that the observed transcriptional changes of mitochondrial biogenesis were not part of the overall change in cellular metabolism. Given that mitochondrial network morphology correlates with mitochondrial biogenesis in response to environmental cues and has recently emerged as a fundamental feature for neuronal and oligodendroglial cell differentiation (Bertholet et al., 2016; Magalon et al., 2016), we examined changes in the mitochondrial network across the different conditions. It was observed that in OPCs exposed to both ENV proteins only or to ENV protein plus folimycin, mitochondria appeared rather fragmented (Figures 6H,J) as compared to buffer-treated (control) cells (Figure 6G). Conversely, OPCs exposed to ENV protein in the presence of either L48H37 or C20 (Figures 6I,K) exhibited more elongated mitochondria. Altogether, these data indicate that mitochondrial shape is affected by ENV protein and can be stabilized *via* L48H37 and C20.

## Discussion

The HERV-W ENV protein has been recognized as an MS-specific pathogenic entity, and functional evidence of its anti-regenerative/remyelinating properties has recently been collected in a clinical trial using the neutralizing antibody GNbAC1/Temelimab (Hartung et al., 2021). Fostering myelin repair in the diseased CNS still represents an unmet clinical need, and it is therefore evident that overcoming the negative impact of ENV can potentially lead to new therapeutic approaches. We here clearly demonstrated that among the three tested TLR4 modulators, L48H37 and C20 exert potent protective/stabilizing effects regarding the severely diminished differentiation and maturation capacity of HERV-W ENV protein challenged oligodendroglial cells, and that this rescue is accompanied by a preserved mitochondrial integrity. Whereas our observation that L48H37 and C20 efficiently inhibit TLR4 surface localization is in accordance with previous studies on macrophages (Wang et al., 2015; Zhang et al., 2016). We could not detect altered surface expression upon folimycin exposure, as demonstrated within macrophages (Eswarappa et al., 2008). Interestingly, L48H37 and C20 were shown earlier to specifically target MD2, which is crucial for glycosylation, translocation, and cell surface expression of TLR4 and its subsequent activation (Ohnishi et al., 2003; Wang et al., 2015; Zhang et al., 2016). Both substances prevent the interaction of TLR4 with MD2 by binding to the hydrophobic region of the MD2 pockets (Wang et al., 2015; Zhang et al., 2016). Thus the formation of the MD2/TLR4 complex fails. But, folimycin was demonstrated in macrophages to alter intra-Golgi pH by inhibiting V-ATPase activity, which in turn results in defective processing and reduced surface expression of TLR4 (Eswarappa et al., 2008). In contrast to the V-ATPase inhibitor KM91104, which specifically targets the a3-b2 subunits of V-ATPase and found to block TLR4 surface expression in oligodendroglial cells (Göttle et al., 2019a), and folimycin binds to the v0 subunit c of V-ATPase, which apparently does not affect the signaling process to the same extent as KM91104 (Huss et al., 2002). In this respect, previous studies revealed the existence of macrophage- and microglia-specific isoforms of the vascular ATPase protein v0 subunit (Peri and Nusslein-Volhard, 2008; Xia et al., 2019), which could indeed result in cell-type specific effects of folimycin.

Hence, a specific blockade of the MD2/TLR4 complex *via* L48H37 or C20 appears more suitable to reduce TLR4 surface expression and for the desired rescue of ENV-mediated oligodendroglial inhibition than the indirect reduction of TLR4 surface expression by using folimycin-related pH alteration through inhibition of V-ATPase, at least in OPCs—so conferring a certain degree of specificity.

Given the limitations that for the human-specific pathogenic HERV-W element yet no suitable *in vivo* model exists the here presented data are limited to examinations *ex vivo*. Further studies on modulated TLR4 signaling in the context of de- and remyelination *in vivo* will be important to conduct and will depend on the generation of a suitable mouse model mimicking HERV-W activation and expression under inflammatory and demyelinating conditions. Moreover, when it comes to future treatment THAT the blood-brain barrier (BBB) still represents the major obstacle when it comes to the delivery of pharmacological substances into the brain parenchyma and its neural cells. L48H37 is an analog of curcumin, a hydrophobic polyphenol extracted from the dried rhizomes of Curcuma longa, with limited BBB penetrance, promising approaches *via* nano delivery of curcumin were recently reported to improve drug delivery (Askarizadeh et al., 2020; Motavaf et al., 2020). C20, on the other hand, as a chalcone derivate of flavonoids/isoflavonoids, is of small molecular size and its lipophilicity suggests a great BBB permeability (Thapa et al., 2021).

It is of considerable interest to note that both substances L48H37 and C20 have already been shown to exhibit protective effects and a certain therapeutic potential for neurodegenerative diseases including Alzheimer’s disease (AD) and Parkinson’s disease (PD) and within demyelinating mouse models, such as experimental autoimmune encephalomyelitis (EAE) and cuprizone-induced demyelination (Darvesh et al., 2012; Feng et al., 2014; Wang et al., 2015; Zhang et al., 2016; Motavaf et al., 2020). Of note, upon cuprizone-induced demyelination, Motavaf et al. (2020) suggested side/pleiotropic effects for curcumin in protecting myelinating cells *via* suppression of astrocytes and microglia.

There is increasing relevance of mitochondrial dysfunction as a major driver in neurodegenerative diseases, such as MS (Barcelos et al., 2019; Mari and Colell, 2021; Tepavcevic, 2021). In this context, also OPC differentiation into myelinating oligodendrocytes leading to myelin repair appears to be a metabolically active process (Kirischuk et al., 1995; Schoenfeld et al., 2010) the disturbance of which can contribute to myelin dysfunction, lack of repair, and neurodegeneration (Barcelos et al., 2019). The dynamic nature of their network structure is crucial for mitochondrial integrity. Mitochondria undergo two opposing processes in that they separate and merge using fission and fusion processes in response to changes in energy and stress status (Liu et al., 2020). The imbalance of this dynamic equilibrium can result in cell injury and contribute to developmental and neurodegenerative disorders (Mari and Colell, 2021). Also excessive oxidative and nitrosative stress can mediate neuronal injury, in part *via* mitochondrial fission or fragmentation. In fact, based on the morphological properties of mitochondria i.e., fission resulting in a shorter length and fewer cristae, it has been suggested to result in decreased ATP synthesis levels in oligodendroglial mitochondria (Rinholm et al., 2016). Our here described findings add to a report of cytotoxic stress within chemo-resistant glioblastoma cells mediated by related HERV-WE_1_ (syncytin-1) and HERV-FRD_1_ (syncytin-2) proteins and shown to trigger large-scale fission of mitochondria (Diaz-Carballo et al., 2017). Consistent with our results, Magalon et al. (2016) demonstrated that a functional interaction between mitochondrial elongation and nitrosative stress exists and contributes to oligodendrocyte maturation, as inhibition of mitochondrial fission/defragmentation led to decreased nitrosative stress levels in OPCs and promoted their maturation.

In addition, gene expression analysis revealed a significant downregulation of genes associated with the peroxisome proliferator- activated receptor gamma (PPAR-γ) signaling pathway upon ENV-protein exposure which could be rescued in presence of L48H37 or C20. Hence, we propose stabilized PPAR-γ signaling as a possible mechanism by which mitochondrial integrity is maintained. This is in accordance with previous studies, pointing to mitochondria as major targets of PPAR-γ signaling shown to protect oligodendroglial cells against nitrosative stress and accelerate oligodendrocyte maturation and maintains mitochondrial function (Bernardo et al., 2009; De Nuccio et al., 2011, 2020). This is further corroborated by the finding that curcumin is able to promote oligodendrocyte differentiation and to protect against nitrosative stress through the activation of the nuclear receptor PPAR-γ (Bernardo et al., 2021).

Collectively, these data highlight two new substances able to counteract the negative impact of HERV-W ENV protein on oligodendroglial homeostasis and suggest mitochondrial fragmentation as an important contributor to blocked OPC differentiation and myelination. Hence, the rescue of mitochondrial homeostasis and morphology following ENV-mediated nitrosative stress induction is a potentially viable and appealing therapeutic strategy in order to promote myelin repair.

## Conclusion

The here presented study has revealed two new substances stabilizing oligodendroglial differentiation in the presence of the HERV-W ENV protein. Moreover, a completely new context regarding ENV-mediated myelination deficits was discovered and unearthed a link to mitochondrial dysfunction. Hence, despite all the efforts made in recent decades regarding myelin repair strategies, mitochondria-targeted compounds with clinical relevance could present an interesting and novel means, indicating that a deeper knowledge of the mechanisms involved within oligodendroglial homeostasis and maturation is still needed. Herein, the curcumin analog L48H37, featuring great chemical stability and enhanced anti-inflammatory properties (Wang et al., 2015), and the small molecule C20 (Zhang et al., 2016), is exciting therapeutic candidate, however, need to be approved for safety, effectiveness, and manufacturing quality in clinical settings.

## Data Availability Statement

The raw data supporting the conclusions of this article will be made available by the authors, without undue reservation.

## Author Contributions

PG and PK contributed to the conception and design of the study, wrote the manuscript, and contributed to funding acquisition. PG, KS, LW, and LR performed the experiments. PG, AZ, KS, and AP contributed to data analysis and interpretation. PG and KS performed the statistical analysis. PG contributed to the data visualization. AP wrote the sections of the manuscript. PK supervised the project. All authors contributed to the article and approved the submitted version.

## Conflict of Interest

PK received consulting, travel, congress grants from GeNeuro SA, Sanofi, and Servier. PG performed consultancy work for GeNeuro SA.

The remaining authors declare that the research was conducted in the absence of any commercial or financial relationships that could be construed as a potential conflict of interest.

## Publisher’s Note

All claims expressed in this article are solely those of the authors and do not necessarily represent those of their affiliated organizations, or those of the publisher, the editors and the reviewers. Any product that may be evaluated in this article, or claim that may be made by its manufacturer, is not guaranteed or endorsed by the publisher.
